# Analysis of Influencing Factors of Cementitious Material Properties of Lead–Zinc Tailings Based on Orthogonal Tests

**DOI:** 10.3390/ma16010361

**Published:** 2022-12-30

**Authors:** Ziyi Yin, Rui Li, Hang Lin, Yifan Chen, Yixian Wang, Yanlin Zhao

**Affiliations:** 1 School of Resources and Safety Engineering, Central South University, Changsha 410083, China; 2 China School of Civil Engineering, Hefei University of Technology, Hefei 230009, China; 3 School of Energy and Safety Engineering, Hunan University of Science and Technology, Xiangtan 411201, China

**Keywords:** lead–zinc tailings, cementitious material, compressive strength, response surface analysis, orthogonal experimental design

## Abstract

At present, the treatment of tailings is mostly carried out in the form of stacking in tailings ponds, resulting in a huge waste of mineral resources and a major threat to the environment and ecology. Using tailings instead of a part of the cement to make cementitious materials is an effective way to reduce the accumulation of tailings. In this paper, lead–zinc tailings-based cementitious materials were prepared by using lead–zinc tailings, fly ash, and ordinary Portland cement, and the effects of four factors on the mechanical properties of lead–zinc tailings, as well as fly ash content, cement content, and water–binder ratio were studied by orthogonal experiments. The corresponding relationship between the factors and the properties of cementitious materials was determined, and the optimization and prediction of the raw material ratio of lead–zinc tailings-based cementitious materials were realized. The test showed the ratio of raw materials to be at the lowest price ratio. Synchronously the ratio that meets the minimum strength requirements was predicted. When the proportion of fly ash:lead and zinc tailings:cement = 30:40:30 and the water–binder ratio was 0.4, the predicted compressive strength of the prepared cementitious material achieved 22.281 MPa, which meets the strength requirements, while the total content of lead–zinc tailings and fly ash was the highest at this time.

## 1. Introduction

The China Mineral Resources Report (2022) states that by the end of 2021, 173 minerals had been discovered in China, including 13 energy minerals, 59 metal minerals, 95 non-metal minerals and six water and gas minerals. Comparing 2020, the new resources increased by 45,597 million tons of coal, 5246 million tons of iron ore, 68,730,900 tons of manganese ore, 7,934,900 tons of copper, and 21,357,800 tons of lead and zinc [1]. With the increasing mining efforts of mineral resources, the production of tailings also increases [2,3], and according to the 2018 National Tailings Storage Data Analysis Report, the tailing reservoir is up to 14,000 and the number remains high.

At present, the construction of tailing ponds for tailings storage is a common method of tailings disposal [4,5]. However, the tailings pond occupies a large amount of agricultural and forestry land and can easily lead to landslides, debris flows, and other geological disasters [6,7,8,9,10]. In addition, the harmful substances formed by heavy metal ions in the tailings pond can also pollute the nearby water and soil resources and affect the normal production and life of the surrounding residents [11,12,13,14]. Tailings storage is not the best method for tailings treatment. Actually, lead and zinc tailings are valuable secondary resources, and a single stockpile does not realize the potential value of their existence, which is also a secondary waste of resources [15,16,17,18]. Therefore, promoting the comprehensive utilization of tailings is significant in increasing resource utilization efficiency [19,20,21], improving environmental quality, and promoting comprehensive green transformation of economic and social development [22,23,24,25]. In recent years tailings have been made into construction materials, chemicals, gelling materials, as well as re-processed tailings together with green and sustainable development using phytoremediation of tailings [26,27,28]. Backfilling the mining area with tailings, not only can bring good economic benefits to the mine, but also can effectively improve the resource utilization rate [29]. It can also reduce the damage to the natural environment and encourage the coordinated development of resources, environment, security, and economy [30]. So in order to solve the problem of the tailing pond accumulation hazard, we actively searched for a lower cost and simpler raw material treatment process on the existing research invention of tailing solid waste utilization to make gelling material [31]. In this paper, the ratio optimization and performance study of lead–zinc tailings as raw material were investigated to make cementitious material to fill the mine.

Most scholars replace part of the clinker with tailings [32,33,34], adding slag, gypsum, and other cementing agents to prepare cementing materials [35,36]. Cascade-grinding has been used to change the gradation of tailings. The strength characteristics of backfill under different tailings gradation were analyzed by using stepwise grinding to change the tailings gradation, and the material ratio which meets the engineering needs was optimized. In addition, through XRD, TG-DTA, IR, and other methods, the changes in the structure of tailings were obtained. Through the design of an iron ore tailings batching test program and firing test research, the optimization ratio of iron tailings blending to burn cementitious materials was successfully completed, and proved that the cementitious materials burned with iron tailings have similar mineral composition and mechanical properties to ordinary silicate cement clinker [37,38].

However, due to the different climatic conditions and geological composition of each situation, the structural composition and chemical composition of each tailings vary greatly [39,40], so the ratio of tailings cementing materials varies from place to place. Regional restrictions require different tailings from different locations to be re-analyzed for chemical composition and structural composition, etc., in order to activate and maintain the optimal ratio. This paper aimed to investigate the preparation of cementitious materials by replacing some cement with lead–zinc tailings through orthogonal tests. The mathematical regression equation of the percentage of each component affecting the compressive strength was established. After conducting regression optimization, influence factor analysis and its related mechanical properties study, the optimal proportion was derived in this paper.

## 2. Test Method

### 2.1. Test Materials

**Figure 1:**
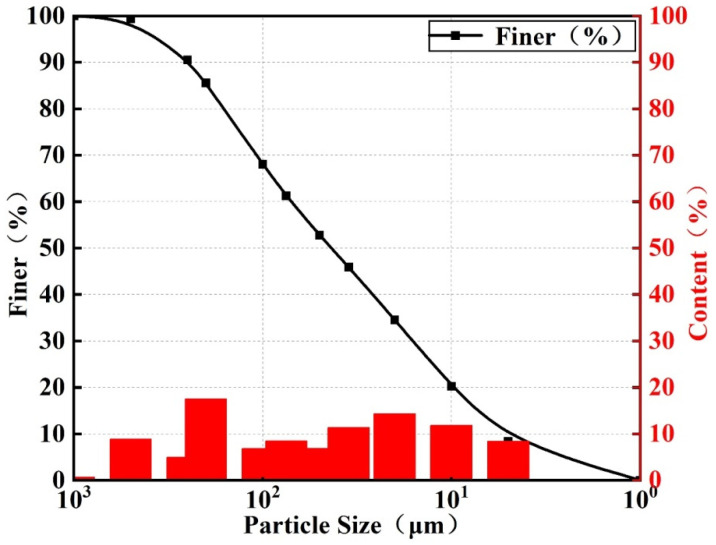
Particle size distribution curve of Pb–Zn tailings.

The test materials included lead–zinc tailings, cement, fly ash, and quartz river sand. The lead–zinc tailings were taken from Sanguikou lead–zinc tailings of Ulat Houqi Zijin Mining Co, Inner Mongolia, China. The tailings particle size is smaller than 1000 microns (Figure 1), The particle size corresponding to the ordinate of 10% and 60% in Figure 1 are d_10_ and d_60_, the inhomogeneity coefficient Cu = d_60_/d_10_ = 15.297 ≥ 5 [41], thus it is well graded and easily mixed and reacted to make cementitious materials. The market price of primary fly ash is 17.244 USD/ton, with a chemical composition similar to clay and a fine particle size. The cement is 425 ordinary silicate cement, whose market price is 54.606 USD/ton. The chemical composition of fly ash and cement is shown in Table 1 [42], and the particle size of river sand was chosen to be uniform and reasonable, with rounded particles, smooth surface, and good fluidity.

**Figure 2:**
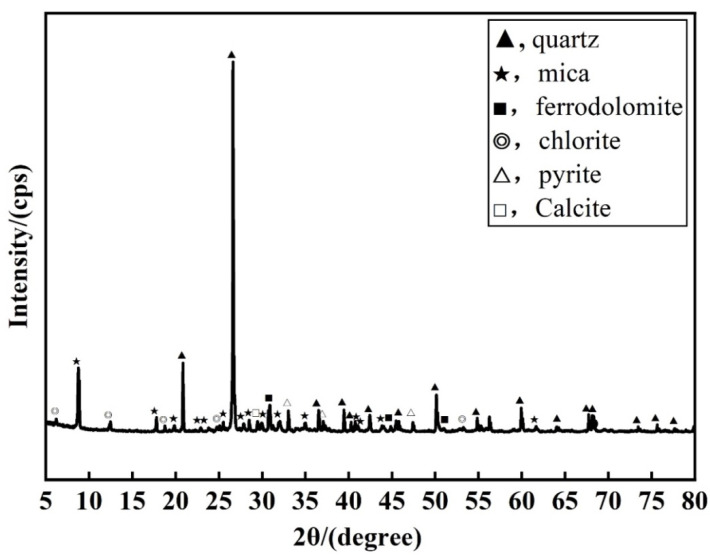
XRD patterns of lead–zinc tailings samples.

In order to determine the mineral composition and chemical composition of lead–zinc tailings, the techniques of X-ray diffraction analysis (XRD) and X-ray fluorescence spectrum analysis (XRF) were used to detect and analyze lead–zinc tailings samples. The X-ray diffraction analysis (XRD) equipment is Bruker D8 ADVANCE, the X-ray fluorescence spectrum analysis (XRF) equipment is Bruker S2 PUMA Series II. The mineral composition analysis results of the tailings are shown in Figure 2, and the chemical composition analysis results are shown in Table 2. The main mineral components of the lead–zinc tailings are quartz, mica, dolomite, chlorite, and they also contain a certain amount of pyrite. The main chemical composition of Pb–Zn tailings is SiO_2_, Fe_2_O_3_, Al_2_O_3_, CaO, and MgO, of which the SiO_2_ content can reach 48.17%. From Table 1 and Table 2, it can be seen that both Pb–Zn tailings and fly ash contain more SiO_2_ compared to cement, therefore, the increase of Pb–Zn tailings and fly ash content is beneficial to the formation of the cementitious material skeleton and improves the denseness of the material. In addition, more active ingredients such as CaO and Al_2_O_3_ in cement and fly ash can maintain the activity of cementitious materials and enhance the cementation of materials [43,44].

### 2.2. Test Design

**Figure 3:**
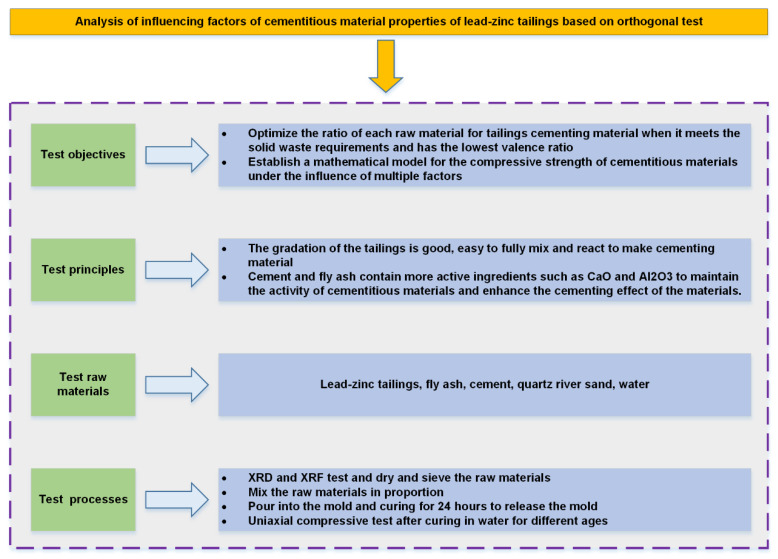
Test Design.

The test design is shown in Figure 3. Lead–zinc tailings and cement were used as the main raw materials, supplemented with a certain amount of fly ash, while river sand was used as the aggregate to prepare the molding test specimens. Two parallel test specimens were made for each set of tests and the average value of the test results taken. The sample involved the ratio design of the raw materials and the change of water–binder ratio, so the orthogonal test method was adopted. This test method is an efficient, rapid, and economical experimental design method, which is often used in material proportioning optimization. The orthogonal test method was used as design in [45,46], and the lead–zinc tailings content, water–binder ratio, and fly ash content were used as test influencing factors. Water has great influence on the mechanical properties of rock body [47,48,49,50], so the water–binder ratio factor test was selected. Each factor was set at four levels, and the selection range of the factor levels was determined by pre-experiment single factor test. The orthogonal test of 3 factors and 4 levels was designed by using the standard orthogonal table L_16_ (4^3^). Sixteen groups of this test were subjected to uniaxial compression test after 3 days, 7 days, and 28 days of maintenance. The factors and their levels corresponding to each group are shown in Table 3.

### 2.3. Test Procedures

**Figure 4:**
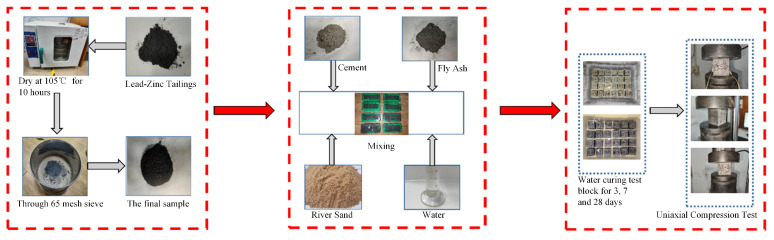
Test procedures.

**Figure 5:**
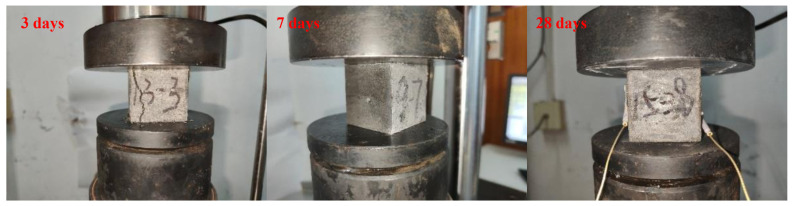
Uniaxial compressive strength test of 3 days, 7 days, and 28 days test block curing.

In the pre-experiment set up a lead–zinc tailings content of 25%40%55% pre-test, it was found that the compressive strength of samples with tailings accounting for 55% is small and the utilization rate of tailings is low. Referring to other scholars’ experiments and research and sample making experience [51,52,53,54], the fly ash variable was increased to take 15% to 30%, the lead–zinc tailings were selected from 25% to 40%, and the water–cement ratio was taken from 0.4 to 0.55, while the three-factor orthogonal test was set at the four levels used, so the range of factors was obtained and then the four levels were set for the formal test design. The factor level range was selected, and the mass ratio of composite cementitious raw material to sand was 1:1.5. During the preparation of the specimens, the impurities in the Pb–Zn tailings were first removed and put into a constant temperature oven at 105 °C for drying treatment, and then removed after 10 h. Then according to ASTM E11, the dried lead–zinc tailing sand was processed through a 60 mesh sieve, while the quartz river sand was processed through a 30 mesh sieve for use. The treated lead–zinc tailings, fly ash, and cement were weighed according to the set proportions and poured into the mixing vessel to mix evenly, after which the corresponding river sand was added and mixed thoroughly. Finally, the corresponding amount of water was added to the mixed material in accordance with the initial water–binder ratio and mixing continued. Moisture was uniformly poured into a 40 mm × 40 mm × 40 mm standard triplex mold gum sand test mold, with vibration molding after covering with cling film and maintaining demolding for 24 h to obtain the test specimens. Subsequently, these samples were immediately immersed in water for maintaining, the test process is shown in Figure 4 The specimens were removed after 3, 7, and 28 days of maintaining respectively, and tested for strength using an electronic pressure tester, the model of the microcomputer-controlled pressure testing machine is HUALONG WHY-300/10, as shown in Figure 5.

## 3. Results and Discussions

### Compression Test Analysis

Compressive strength is often used as a standard to determine the mechanical properties of materials [55,56,57]. Many scholars have used model research [47,58,59,60,61,62], this paper was based on the response surface method to fit the mathematical equation. Sixteen groups of this test were subjected to uniaxial compressive strength test after 3 days, 7 days, and 28 days of maintaining. The test results are shown in Table 4.

The results of the analysis of the strength of the lead–zinc tailings cementitious material under different levels of each influencing factor are shown in Figure 5, and the factor levels are the contents of the factors from low to high, as shown in Table 5.

**Figure 6:**
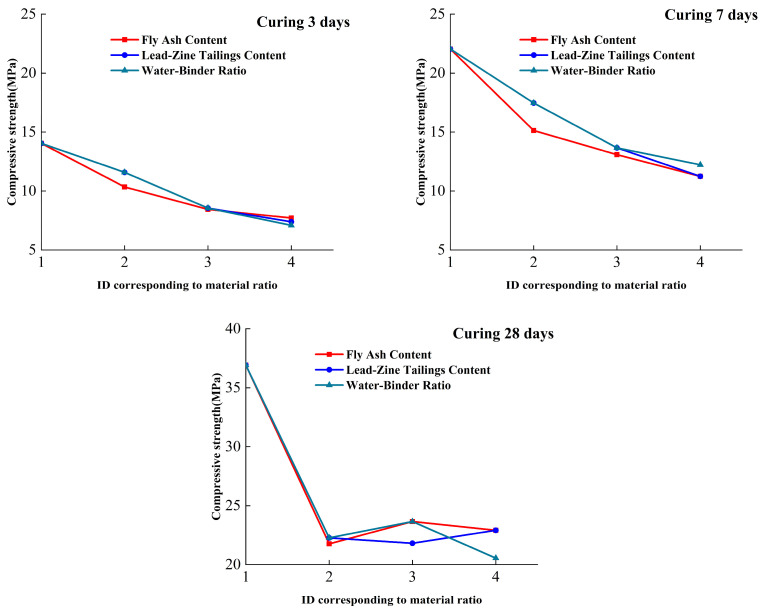
The change diagram of compressive strength at 3 days, 7 days, and 28 days curing of each factor.

From Figure 6, it can be seen that, by extreme difference comparison, the size of the sensitivity of each factor on strength at 3-day age is water–binder ratio > lead–zinc tailings content > fly ash content. However, the extreme difference in compressive strength of the water–binder ratio factor with the highest sensitivity at four different levels is 6.96 MPa, and the extreme difference in compressive strength of the fly ash factor with the lowest sensitivity is 6.33 MPa, so the difference between the three effects is not large. The compressive strength is up to 14.04 MPa at factor level 1, and the lowest is 3 MPa at factor level 4. In the subsequent application, the water–binder ratio can be used as an important influencing factor for the first choice when the curing age is three days.

The sensitivity of each factor to strength at 7-day age is fly ash content > lead–zinc tailings content > water–binder ratio. Although the range of the first two factors is the same, the fly ash content fluctuates greatly at different levels and is more sensitive. The compressive strength reached a maximum of 22.04 MPa at factor level 1 and a minimum of 5.08 MPa at factor level 4. Compared with the 3-day age, the sensitivity of the water–binder ratio becomes smaller, indicating that as the curing time becomes longer, each factor gradually undergoes a hydration reaction.

The sensitivity of each factor to strength at 28-day age is water–binder ratio > lead–zinc tailings content > fly ash content. The maintenance time is sufficient, and the hydration reaction is further accelerated. The compressive strength reached a maximum of 36.92 MPa at factor level 1, and a minimum of 21.76 MPa at factor level 4. The water–binder ratio eventually became the most important factor affecting the later gelation performance.

The compressive strength increased with the growth of maintaining days under the influence of different factor levels. The best curing period was 28 days among the three, and the compressive strength was significantly improved.

## 4. Response Surface Prediction Regression Analysis

### 4.1. Regression Analysis

In order to explore the specific effects of the sensitivity of each factor on the compressive strength of the gelling material, regression equation analysis of the uniaxial compressive strength data of the test blocks was conducted. Response surface regression analysis of uniaxial loading strength affected by three factors under different curing periods was carried out by mathematical analysis software. In all regression equations, from Equations (1)–(3), A, B, and C represent the content of fly ash, lead–zinc tailings, and water–binder ratio respectively. To fully validate the model accuracy, the validation group should reflect the variation range of each factor. Thus, groups numbered 1, 7, 12, and 14 were selected to verify the accuracy of the model, and the other 12 groups were used as the basic data for model building.

After inputting the result setting parameters, the regression equation for the strength of the 3-day cured test block was derived:

(1) $$y=57.005-2.63A-0.311B-9.348C+1.634\mathrm{AC}-1.742\mathrm{BC}+0.035A^{2}+0.013B^{2}$$

The results of the 3-day conservation regression model ANOVA (Table 6) showed that the model significance test *p* = 0.0328 < 0.05 and the model fitting ability were good, where the corrected coefficient of determination R2 = 0.9316 > 0.80 was statistically significant.

Regression equation for strength of 7-day curing specimens:

(2) $$y=85.157-2.889A-1.413B-2.631C+0.043\mathrm{AB}-1.139\mathrm{AC}+0.034A^{2}$$

From the results of the ANOVA of the 7-day maintenance age regression model (Table 7), it can be seen with the model significance test *p* value = 0.0114 < 0.05, where the corrected coefficient of determination R2 = 0.9234 > 0.80, that the model fitting ability is good and statistically significant.

Regression equation for strength of 28-day curing specimens:

(3) $$\;y=671.63609-0.569A-40.714B-1206.267C+78.71\mathrm{BC}+0.667B^{2}-1.328B^{2}C$$

From the results of the ANOVA of the 28-day maintenance age regression model (Table 8), it can be seen that the model *p*-value = 0.0214 < 0.05 and the equation has good fitting ability, where the corrected coefficient of determination R2 = 0.9003 > 0.80 is statistically significant and the regression equation is available.

**Figure 7:**
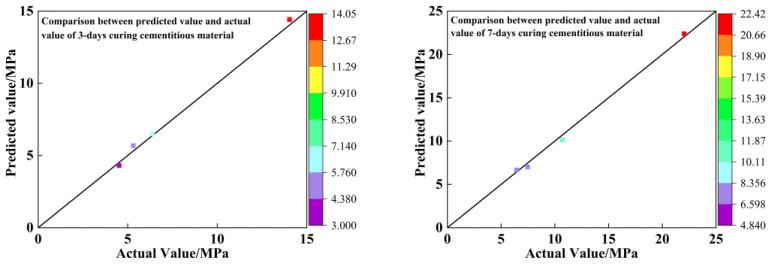

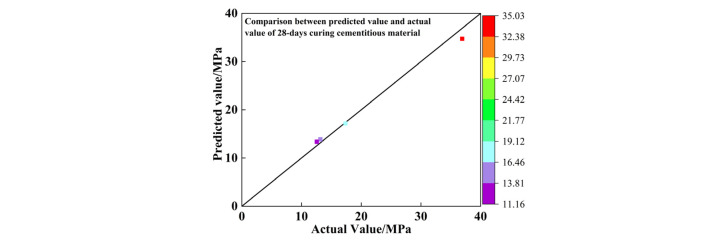
Comparison of predicted and actual values of compressive strength for different curing days.

The R^2^ values of the complex correlation coefficients of the regression equations are all greater than 0.90, indicating that the equations fit well and can predict the strength of the filler at each age more accurately [63,64,65]; the three different maintenance period strength results in the regression equation, the *p* value response of the ABC three factors, is the degree of influence on compressive strength. From Table 6, Table 7 and Table 8, it can be seen that all three factors have a significant effect on the compressive strength. The errors between the predicted and actual values of the compressive strength equation of the cementitious material are shown in Table 9. To show the relationship between actual and predicted values more visually, Table 9 was transformed into the one shown in Figure 7. From Figure 7, it is clear that the predicted values of compressive strength of the gelling material are close to the actual values on the y = x straight line, which further proves that this regression equation fits well.

**Figure 8:**
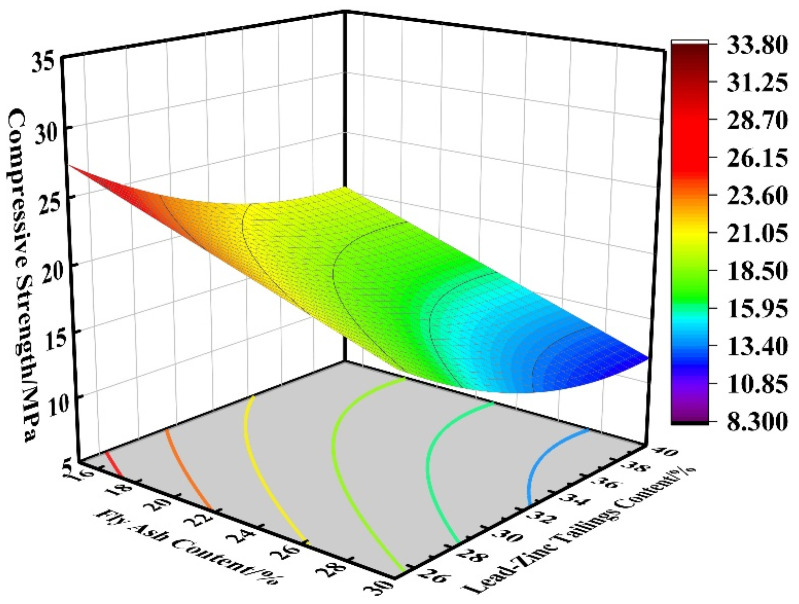
Effect of AB on compressive strength.

Since most of the tailings gelling specimens cured for 28 days reached the maximum strength and the hydration reaction was sufficient, the 28-day curing age gelling specimens were selected for the optimized proportioning and performance study. When the water–binder ratio is 0.48, the effects of fly ash and lead–zinc tailings on the compressive strength of cementitious materials are shown in Figure 8. In this study, fly ash content and lead–zinc tailings content are used as independent variables and compressive strength is the dependent variable. It can be seen from the figure that with the increase of fly ash content and lead–zinc tailings content, the compressive strength of 28-day curing specimens gradually decreases. The highest compressive strength is reached at 15% fly ash content and 25% tailings content; The compressive strength reaches its minimum at 30% fly ash content and 40% tailings content. In the range of test factor levels, when the fly ash content is constant, the higher the lead–zinc tailings content, the lower the compressive strength, and in a certain range, with about every 2% increase in lead–zinc tailings content, the strength of the gel test block is reduced by 2 MPa; When the content of lead–zinc tailings is constant, the greater the fly ash content, the smaller the compressive strength, in a certain range; for approximately every 4% increase in fly ash content, the strength of the gelling test block decreases by 2 MPa. This again demonstrates that the sensitivity of the Pb–Zn tailings content is greater than the sensitivity of the fly ash content. For 28-day age cementitious material, the tailings content of 20% to 30% with fly ash content of 15% to 22% is selected to facilitate a substantial increase in compressive strength.

**Figure 9:**
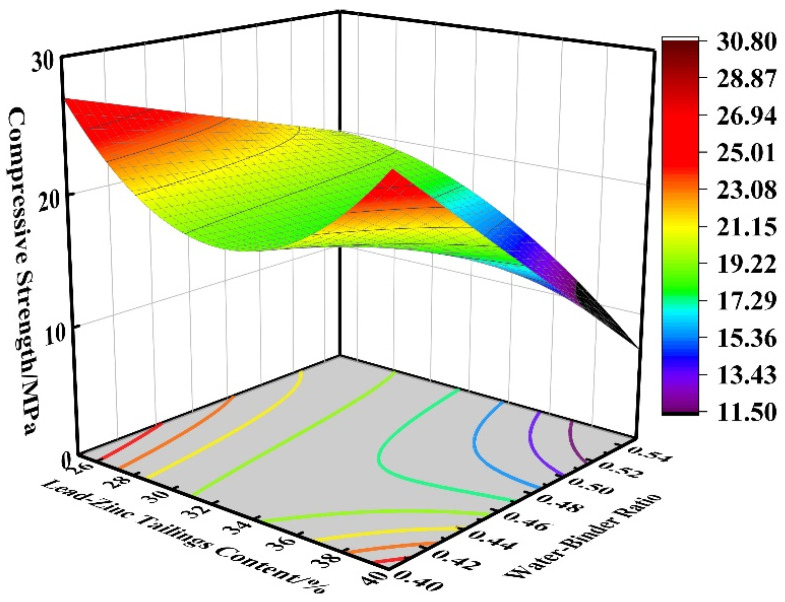
Effect of BC on compressive strength.

At a fly ash content of 22.5%, the effects of lead–zinc tailings content and water-cement ratio on the compressive strength of the cementitious material are shown in Figure 9. The highest compressive strength is reached when the lead–zinc tailings content is 25% and the water–binder ratio is 0.4; The transition in color of the surfaces in the figure signifies the variation of different compressive strengths. In the xy plane, the rightmost contour is the minimum value of compressive strength. When the content of lead–zinc tailings is 40% and the water–binder ratio is 0.47, the compressive strength is the lowest. When the content of lead–zinc tailings is 25–35%, it has a great influence on the compressive strength, which is the main position and has a negative correlation, while the influence of water–binder ratio is secondary. However, when the content of lead–zinc tailings is high, at 35–40%, the compressive strength is mainly affected by the water–cement ratio. When the water–binder ratio is in the range of 0.47–0.55, the higher the content of lead–zinc tailings, the lower is the compressive strength. In a certain range, about every 2% increase in lead–zinc tailings content, compressive strength decreases by 3 MPa; in the water–binder ratio in the range of 0.4–0.47, the compressive strength decreases first and then increases with the increase of lead–zinc tailings in an upward parabola, with the rightmost contour corresponding to the strength taking the minimum value. When the content of lead–zinc tailings is constant, the compressive strength reduces with the increase of water–binder ratio. In a certain range, the water–binder ratio increases about every 0.01, the compressive strength decreases by 2 MPa.

### 4.2. Optimized Ratios

Optimal proportioning can be considered using two different methods. The first one calculates the cost of one ton of raw materials for each test grouping, based on the cost of the market price of the materials used in the test. As shown in Table 10, the cost is divided by the corresponding compressive strength at different times to obtain the price ratio corresponding to the test grouping. It can be calculated that if the price ratio is smaller, the raw material utilization rate is higher and the cost is smaller. In addition, according to the “T/CECS 689-2020-Technical specification for application of solid waste cementitious material”, the general cementitious strengths of 3-day and 28-day solid waste-based cementitious materials are as shown in Table 11. As can be seen from the table, in the test to achieve the strength requirements at 3 days of age and 28 days of age, the lowest price ratio at 3 days of age is the first group of tests, and the lowest price ratio at 28 days of age is also the first group of tests. At this time, the water-cement ratio is 0.4, the lead–zinc tailings content is 25%, the fly ash content is 15%, and the cement content is 60%.

In the optimized ratio selection, the second method takes into account the market price of raw materials, the specific manipulability of the test, and the uniaxial compressive strength requirements of the test block of cementitious materials, and sets the content of lead–zinc tailings > fly ash > cement in the numerical analysis software according to the price from low to high. Compressive strength up to 4 MPa as predicted by Equation (1) is chosen for the water–binder ratio of 0.49, with lead–zinc tailings content of 40%, fly ash content of 30%, and cement content of 30%. When the water–binder ratio is 0.40, the content of lead–zinc tailings is 29.12%, the content of fly ash is 22.56%, and the content of cement is 48.32%, the compressive strength is predicted to reach 15 MPa by Equation (2). When the water–cement ratio is 0.4, lead–zinc tailings content is 40%, fly ash content is 30%, and cement content is 30%, the compressive strength is predicted by Equation (3) to reach 22.281 MPa, which satisfies the requirements and reaches the highest cost performance ratio at this time.

## 5. Conclusions

In this paper, a 3-factor, 4-level, 16-group orthogonal test was designed in parallel for 3 days, 7 days, and 28 days for the maintenance of cementitious materials made from Sanguikou lead–zinc tailings in Inner Mongolia. Based on orthogonal tests, the factors influencing the performance of lead–zinc tailings cementitious materials were analyzed. A mathematical regression analysis of uniaxial compressive test results was carried out by numerical analysis software, and the mathematical relationship between factor ratios and compressive strength under different maintenance days was derived. The conclusions are as follows:

(1) The lead–zinc tailings are well graded and contain mainly quartz, mica, dolomite, chlorite, and other mineral components. The main chemical components are Fe_2_O_3_, SiO_2_, Al_2_O_3_, MgO, CaO, etc.
(2) The sensitivity of each factor to strength at 3 days of age is water–binder ratio > lead–zinc tailings content > fly ash content; The sensitivity of each factor to strength at 7 days of age is fly ash content > lead–zinc tailings content > water–binder ratio; The sensitivity of each factor to strength at the age of 28 days is water–binder ratio > lead–zinc tailings content > fly ash content. For specimens under a short curing period (3 d), the most powerful sensitivity parameter is water–binder ratio. The best curing period for specimens is 28 d. With sufficient hydration, the strength is significantly higher than that of the specimen with curing periods of 3 d and 7 d.
(3) For the comprehensive realistic price factors and compressive strength requirements of cementitious materials in the known test group, a water–binder ratio of 0.4 is chosen for the 28-day age cementitious material, and the ratio of fly ash:lead–zinc tailings:cement = 30:40:60, when the valence ratio is 0.38 USD/MPa. In the equation prediction, fly ash:lead–zinc tailings:cement = 30:40:30, with the water–binder ratio of 0.4 is the optimal ratio, when the compressive strength can reach 22.281 MPa.

The compressive strengths of the cementitious materials prepared by high-temperature heat activation of lead–zinc tailings increase but due to the temporary unavailability of the test equipment, the specimens were prepared only at room temperature without considering the effect of temperature on the properties of the cementitious materials. In subsequent study, the effect of thermal activation temperature and holding time on the properties of lead–zinc tailings gelling materials will be investigated.

## Figures and Tables

**Table 1:** Chemical compositions of fly ash and cement.

Material	Chemical Compositions (%)							
	Na_2_O	SiO_2_	Al_2_O_3_	MgO	CaO	P_2_O_5_	K_2_O	Fe_2_O_3_
Fly Ash	1.670	48.800	26.260	1.840	4.951	0.146	2.000	4.869
Cement	0.276	14.240	5.410	1.799	52.84	0.408	0.892	2.461

**Table 2:** Chemical compositions of lead–zine tailings.

Material	Chemical Compositions (%)										
	TFe	SiO_2_	Al_2_O_3_	MgO	CaO	K_2_O	MnO_2_	TiO_2_	Na_2_O	ZnO	Other
Lead–Zine Tailings	14.15	48.17	10.79	4.14	4.20	3.01	0.73	0.31	0.46	0.49	13.55

**Table 3:** Mix proportions of experiment.

Sample No.	Fly Ash/%	Lead–Zinc Tailings/%	Cement/%	Water–Binder Ratio
1	15	25	60	0.4
2	15	30	55	0.45
3	15	35	50	0.5
4	15	40	45	0.55
5	20	25	55	0.55
6	20	30	50	0.4
7	20	35	45	0.45
8	20	40	40	0.5
9	25	25	50	0.5
10	25	30	45	0.55
11	25	35	40	0.4
12	25	40	35	0.45
13	30	25	45	0.45
14	30	30	40	0.5
15	30	35	35	0.55
16	30	40	30	0.4

**Table 4:** Compressive strength of different curing days (MPa).

Sample No.	3 Days	7 Days	28 Days
1	14.04	22.04	36.92
2	11.58	17.46	22.27
3	8.55	13.66	21.81
4	5.3	8.5	12.16
5	7.09	12.22	20.55
6	10.34	15.13	21.76
7	6.39	10.71	17.36
8	4.21	6.01	12.82
9	8.44	13.08	23.65
10	5.29	7.85	15.41
11	6.02	9.44	17.91
12	4.52	6.45	13.16
13	7.71	9.22	19.25
14	5.31	7.47	12.56
15	3	5.08	10.59
16	7.39	11.24	22.91
Mean value	7.20	10.97	18.82
Standard deviation	2.81	4.39	6.30

**Table 5:** Orthogonal test factors and levels.

Levels	Lead–Zine Tailings (%)	Fly Ash(%)	Water–Binder Ratio
1	25	15	0.40
2	30	20	0.45
3	35	25	0.50
4	40	30	0.55

**Table 6:** Variance of 3-day curing cementitious material compressive strength model.

Source of Variation	Mean Square	Degrees of Freedom	Quadratic Sum	Value *p*
Model	63.05	7	9.01	0.0328
A	21.17	1	21.17	0.0129
B	14.36	1	14.36	0.0244
C	25.35	1	25.35	0.0094
AC	0.91	1	0.91	0.4259
BC	2.19	1	2.19	0.2407
A^2^	6.55	1	6.55	0.0760
B^2^	0.56	1	0.56	0.5247
Residual	4.63	4	1.16	
SUM	67.68	11		

**Table 7:** Variance of 7-day curing cementitious material compressive strength model.

Source of Variation	Mean Square	Degrees of Freedom	Quadratic Sum	Value *p*
Model	140.84	6	23.47	0.0114
A	71.81	1	71.81	0.0026
B	54.05	1	54.05	0.0048
C	26.96	1	26.96	0.0193
AB	16.61	1	16.61	0.0446
AC	1.12	1	1.12	0.5190
A^2^	7.97	1	7.97	0.1241
Residual	11.69	5	2.34	
SUM	152.53	11		0.0114

**Table 8:** Variance of 28-day curing cementitious material compressive strength model.

Source of Variation	Mean Square	Degrees of Freedom	Quadratic Sum	Value *p*
Model	208.41	6	34.74	0.0214
A	54.38	1	54.38	0.0186
B	59.47	1	59.47	0.0157
C	3.53	1	3.53	0.4218
BC	27.52	1	27.52	0.0586
B^2^	11.61	1	11.61	0.1737
B^2^C	20.44	1	20.44	0.0893
Residual	23.09	5	4.62	
SUM	231.50	11		

**Table 9:** Residual value of compressive strength at different curing time.

	3 Day Age/MPa			7 Day Age/MPa			28 Day Age/MPa		
Test Group Number	Actual Value	Predicted Value	Error Magnitude	Actual Value	Predicted Value	Error Magnitude	Actual Value	Predicted Value	Error Magnitude
1	14.04	14.42	0.38	22.04	22.38	0.34	36.92	34.72	−2.2
7	6.39	6.50	0.11	10.71	10.12	−0.59	17.36	17.15	−0.21
12	4.52	4.31	−0.21	6.45	6.66	0.21	13.16	13.85	0.69
14	5.31	5.68	0.37	7.47	7.00	−0.47	12.56	13.36	0.8

**Table 10:** Price ratio of different curing times.

Test Group Number	Test Cost (USD/ton)	3 d Price Ratio (USD/MPa)	7 d Price Ratio (USD/MPa)	28 d Price Ratio (USD/MPa)
1	14.14	1.01	0.64	0.38
2	15.91	1.37	0.91	0.71
3	17.68	2.07	1.29	0.81
4	19.44	3.67	2.29	Strength does not match
5	19.44	2.74	1.59	0.95
6	14.14	1.37	0.93	0.65
7	15.91	2.49	1.49	Strength does not match
8	17.68	4.20	2.94	Strength does not match
9	17.68	2.09	1.35	0.75
10	19.44	3.68	2.48	Strength does not match
11	14.14	2.35	1.50	Strength does not match
12	15.91	3.52	2.47	Strength does not match
13	15.91	2.06	1.73	Strength does not match
14	17.68	3.33	2.37	Strength does not match
15	19.44	Strength does not match	3.83	Strength does not match
16	14.14	1.91	1.26	0.62

Raw material market price: Grade I fly ash 17.24 USD/ton, 42.5 Portland cement 54.61 USD/ton.

**Table 11:** General sand strength of solid waste cementitious materials.

Strength	Grade	Compressive Strength	
General mortar strength		3 d	28 d
	I	≥4.0	≥20.0

## Data Availability

Some or all data, models, or code that support the findings of this study are available from the corresponding author upon reasonable request.
